# Impact of Activation Functions on the Detection of Defects in Cast Steel Parts Using YOLOv8

**DOI:** 10.3390/ma18122834

**Published:** 2025-06-16

**Authors:** Yunxia Chen, Yangkai He, Yukun Chu

**Affiliations:** 1 School of Intelligent Manufacturing and Control Engineering, Shanghai Polytechnic University, Pudong District, Shanghai 201209, China; 20241513125@sspu.edu.cn (Y.H.); 15156237832@163.com (Y.C.); 2 School of Materials Science and Engineering, Shanghai Jiao Tong University, Minhang District, Shanghai 200240, China

**Keywords:** activation function, YOLOv8, gradient-weighted class activation mapping (Grad-CAM), deep learning

## Abstract

In this paper, to address the issue of the unknown influence of activation functions on casting defect detection using convolutional neural networks (CNNs), we designed five sets of experiments to investigate how different activation functions affect the performance of casting defect detection. Specifically, the study employs five activation functions—Rectified Linear Unit (*ReLU*), Exponential Linear Units (*ELU*), *Softplus*, Sigmoid Linear Unit (*SiLU*), and *Mish*—each with distinct characteristics, based on the YOLOv8 algorithm. The results indicate that the *Mish* activation function yields the best performance in casting defect detection, achieving an mAP@0.5 value of 90.1%. In contrast, the *Softplus* activation function performs the worst, with an mAP@0.5 value of only 86.7%. The analysis of the feature maps shows that the *Mish* activation function enables the output of negative values, thereby enhancing the model’s ability to differentiate features and improving its overall expressive power, which enhances the model’s ability to identify various types of casting defects. Finally, gradient class activation maps (Grad-CAM) are used to visualize the important pixel regions in the casting digital radiography (DR) images processed by the neural network. The results demonstrate that the *Mish* activation function improves the model’s focus on grayscale-changing regions in the image, thereby enhancing detection accuracy.

## 1. Introduction

With the advancement of industrialization, industrial castings have become an integral part of daily life. From home furnishings to travel equipment, these products rely heavily on industrial castings. The quality of such castings directly and indirectly impacts people’s lives. Precision castings, in particular, serve as critical components in large machinery and are widely utilized across various sectors [1]. Especially in transportation systems such as railroads and aerospace, the quality requirements for casting products are stringent [2], as the safety of human lives depends on the integrity of these components. Therefore, ensuring the quality of castings through rigorous testing is crucial before they leave the factory. The primary factors affecting casting quality include the manufacturing process, casting structure, and raw material quality, all of which can lead to a variety of defects within the castings [3]. Nondestructive testing (NDT) [4] techniques, including radiographic testing [5], ultrasonic testing [6], and eddy current testing [7], can help identify these defects. Among these methods, radiographic nondestructive evaluation technology is widely used due to its high-resolution imaging capabilities. Currently, the detection of casting defects primarily relies on human inspection of digital radiography (DR) images obtained from X-ray scans; however, this method has low efficiency and is prone to variability based on the inspector’s condition [8,9].

In recent years, artificial intelligence has been advancing across various fields, offering innovative solutions to problem solving, and the detection of defects is no exception. In the domain of casting defect detection, numerous deep learning-based methods have emerged [10]. Jiang Lili et al. [11] introduced a weakly supervised convolutional neural network (CNN) for defect recognition using casting X-ray images. By employing weak supervision on each image, an attention map is generated to highlight defective regions. The model further refines its focus on discriminative features through a combination of intercommunication loss and cross-entropy loss, achieving a recall rate of 96.0%. Du Wangzhe et al. [12] developed an advanced deep defect detection model specifically designed for X-ray images of automotive aluminum castings. This model utilizes Faster R-CNN as its backbone and incorporates a feature pyramid network to enhance its multiscale detection capabilities. Additionally, the introduction of the convolutional block attention module (CBAM) attention mechanism significantly improves the network’s proficiency in identifying defect areas. As a result, the model has achieved an impressive average detection accuracy of 93.7%. Tang Zhenhui et al. [13] proposed a nondestructive testing method for improving the defect detection accuracy on X-ray images of castings. By incorporating bilinear convolutional neural networks and spatial attention mechanisms, the network’s capability to extract and differentiate complex features has been significantly improved, leading to enhanced defect detection accuracy for small targets. Shao Qiufeng et al. [14] developed a detection model known as FFDR-Net, which is based on an enhanced version of RetinaNet. They incorporated ResNet-152 as the backbone to improve the network’s feature extraction capabilities. Building upon the feature pyramid network, they introduced a feature fusion module aimed at enhancing the detection accuracy for small targets. As a result, the average detection accuracy of the improved model increased by 6.6%. Yongxiong Wang et al. [15] developed a novel deep learning model for detecting subtle defects in aluminum alloy castings from DR images. This model integrates a general feature network (GFN) with a subtle feature network (SFN), incorporating a self-attention mechanism within a self-attention guided module (SGM) in the SFN. This approach enhances the model’s capability to extract subtle features in complex backgrounds, resulting in superior detection performance. All these methods improve detection accuracy by enhancing the network’s ability to concentrate on target features. These advancements are specifically applied to DR images obtained via X-ray technology.

Although significant effort has been devoted to the detection of defects in casting DR images, several challenges remain. First, casting defects often exhibit pixel values that are very close to those of the surrounding areas on DR images. Second, these defects are typically characterized by their small size [16]. Third, certain types of porosity and inclusion defects have similar appearances, making them difficult to distinguish. These three factors collectively complicate the detection process for models. In convolutional neural networks (CNNs), the input is the pixel values of the image, which are processed layer by layer through operations such as convolution, regularization, and activation to extract key information. Convolution is used for feature extraction, regularization addresses the internal covariate shift problem [17], and the activation function introduces nonlinearity, which is crucial for enhancing the expressiveness of the neural network [18]. Different activation functions play distinct roles due to their unique characteristics. Vasanthakumari et al. [19] designed Modified Rectified Linear Unit (MReLU) to allow for the absolute value of small negative gradients, thereby preserving important information present in the negative values of the convolutional layers and introducing higher nonlinearity to the output, leading to improved detection performance. Wu KeZhu et al. [20] substituted the original sigmoid linear unit (*SiLU*) activation function in the model with the rectified linear unit (*ReLU*) activation function. This modification not only maintained detection accuracy but also reduced computational complexity, accelerated processing speed, and significantly enhanced stability. Yi Weiguo et al. [21] conducted defect detection on insulators; they compared three different activation functions. Ultimately, they substituted the *SiLU* function with the *Mish* function, which effectively mitigated image distortion and enhanced detection accuracy. Hu Ande et al. [22] conducted a study on defect detection in welding utilizing convolutional neural networks. They discovered that the exponential linear unit (*ELU*) activation function significantly enhanced the model’s robustness against varying characteristics present in different input images, while also improving both the convergence speed and the stability of the model. Adem et al. [23] evaluated various activation functions in a CNN model for detecting DR lesion exudates and found that the Swish function outperformed other activation functions. Mondal et al. [24] proposed a new parametric neural network model called Parametric Flatten-p *Mish* (PFpM) for classifying brain tumors in CNNs. This new parametric activation function addresses neuronal death and bias transfer effects, achieving high classification accuracy across different datasets.

For casting defects, their inherent characteristics result in the feature values extracted by convolution for different types of defects having very small differences, leading to weak discriminative ability in the model. The activation function not only introduces nonlinear factors but also serves as the input for the next layer of neurons, thereby determining the feature values of subsequent layers. Consequently, the nonlinear mapping output of the activation function is particularly crucial for the detection performance of the model, especially when the differences in input feature values are minimal.

For certain deep learning models, a relatively high detection accuracy can be achieved when addressing complex defect features or sparse targets. However, current research in industrial detection primarily focuses on enhancing feature extraction modules and model loss functions, while the critical role of activation functions within these models is underexplored. Consequently, to address this gap, the aim of this study is to apply various activation functions to convolutional neural networks (CNNs) and analyze their properties and effects on casting defect detection. We selected five activation functions with distinct characteristics for the experiment, analyzed the mean and variance at different network layers, and utilized the regions of interest from the Grad-CAM visualization model in the small object detection layer. Its primary contributions are summarized as follows:

1. By analyzing five types of activation functions—*ReLU*, *ELU*, *Softplus*, *SiLU*, and *Mish*—and their corresponding curve graphs, we systematically elucidated the inherent advantages and disadvantages of each type of activation function.

2. To evaluate the impact of different activation functions on the model’s detection performance, experiments were conducted comparing five models on the test set. The results demonstrate that the *Mish* activation function exhibits superior detection performance, enhancing both the mapping capability and the recognition ability of the model with respect to target features.

3. By comparing the effects of the five activation functions across various feature layers and statistically analyzing their means and variances, it is concluded that the *Mish* activation function enhances the model’s ability to differentiate features, thereby improving its expressive power.

4. Through gradient-based class activation mapping, it is shown that the *Mish* activation function improves the model’s ability to focus more effectively on areas of gray-level variation in images, thereby enhancing the model’s detection accuracy.

The remainder of this article is organized as follows: Section 2 discusses the characteristics of the dataset, explains the principles of the YOLOv8 algorithm, and provides a detailed analysis of various activation functions. Section 3 presents an in-depth examination of the experimental results and discussions, including the impact of different activation functions on the validation and test sets, an exploration of mean and variance across distinct feature layers, and a comparative analysis using gradient-based class activation mapping. Finally, Section 4 summarizes the research conclusions.

## 2. Methodology

### 2.1. Dataset

This study utilized a dataset of 6293 digital radiographic images of cast steel parts from Qingdao Sanheshan Precision Casting Co., Ltd. (Qingdao, China). Castings were produced through sand casting. Their images were captured using X-rays, resulting in digital radiographic images. The collected images were preprocessed. All experimental images were resized to 640 × 640 pixels, and their visual quality was enhanced via an image enhancement algorithm. The dataset was manually created and reviewed by qualified technicians. The dataset encompassed three common types of casting defects: 5562 instances of porosity defects, 9508 instances of inclusion defects, and 2850 instances of loose defects. The dataset was divided into training, validation, and test sets according to the ratio of 16:4:5 for experimental purposes.

**Figure 1:**
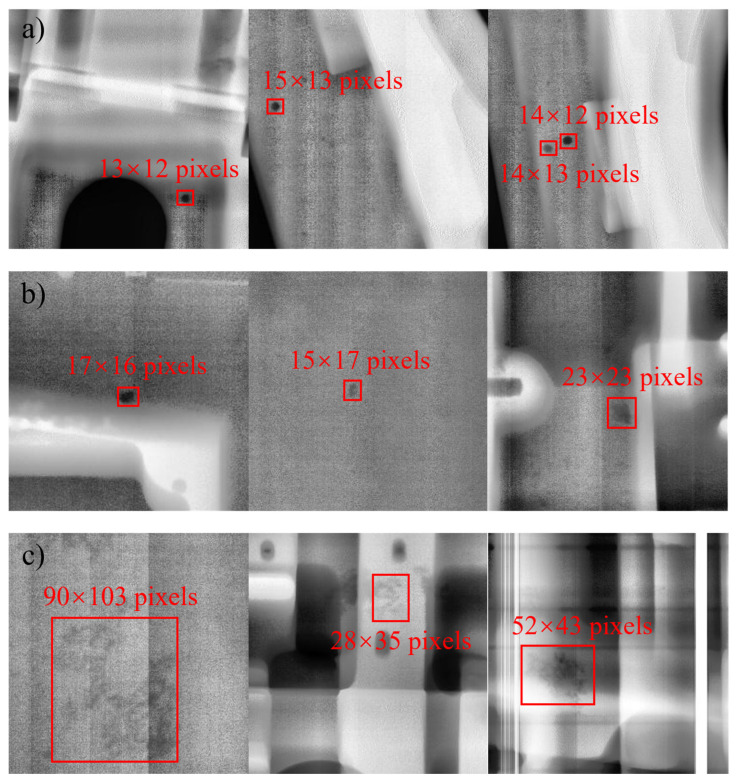
Digital Radiography (DR) images of casting defects: (**a**) porosity, (**b**) inclusion, (**c**) looseness.

**Figure 2:**
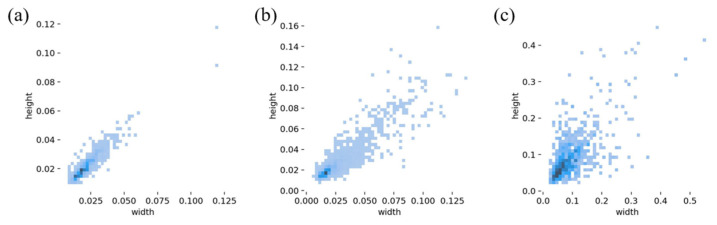
The proportion of casting defects sizes: (**a**) porosity, (**b**) inclusion, (**c**) looseness.

Figure 1 presents the DR images of casting defects. Specifically, porosity defects typically arise when gas trapped in the liquid metal during pouring cannot be promptly expelled, leading to internal voids that appear as round or oval shapes on the DR image. Inclusion defects occur when residual slag enters the mold cavity prior to pouring, forming irregular polygonal structures visible on the DR image. Lastly, loose defects result from non-uniform cooling during solidification, causing localized insufficient shrinkage and manifesting as sponge-like or dendritic patterns on the DR image. The differences among similar defects are primarily reflected in pixel size, as illustrated in Figure 2.

Figure 2 illustrates the ratio of the labeled size of casting defects in the dataset to the size of the original image. As shown in the figure, both porosity and inclusion defects have relatively small labeled sizes, typically occupying less than 10% of the original image size. In the context of target detection, these are classified as small targets, making them more challenging for the model to detect. By contrast, loose defects exhibit a relatively larger labeled size, which makes them easier for the model to identify.

### 2.2. YOLOv8 Model

**Figure 3:**
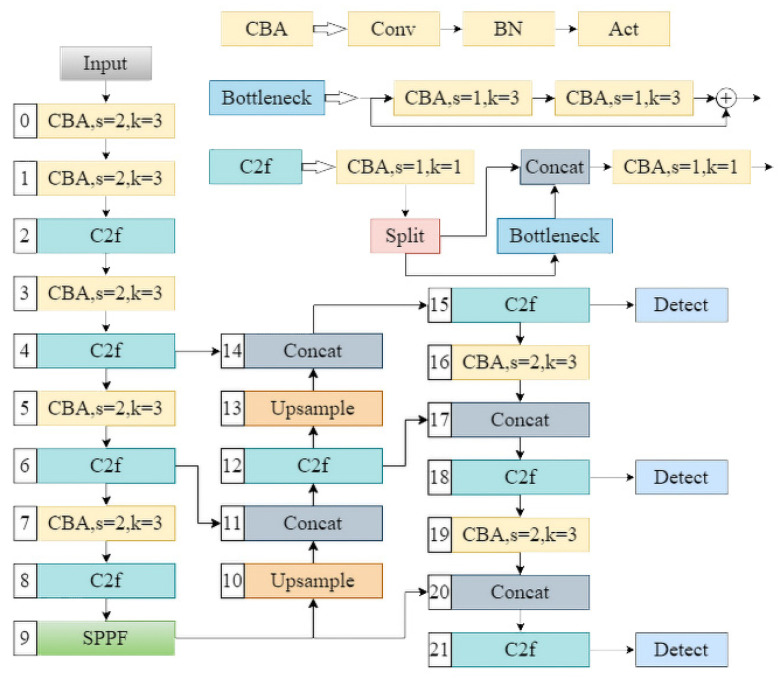
YOLOv8 frame diagram.

This study is conducted based on the YOLOv8 model [25], and the network structure is illustrated in Figure 3. The activation function is defined after each convolution and regularization step. The other components of the model remain unchanged while the experiments are conducted using various activation functions. In each experiment, we utilized the Ultralytics framework to modify the activation functions employed by the model. For this study, the input casting image size is set to 640 × 640 pixels, which is progressively compressed through several modules. Simultaneously, the number of feature channels increases gradually from 3 channels in the input image to 1024 channels in the deepest layer. Each feature layer contains critical information about the casting, influencing the prediction results. The model includes three detection heads for the feature maps at layers 15, 18, and 21, respectively.

### 2.3. Activation Functions

Activation functions play a crucial role in introducing nonlinear fitting capabilities to neural networks. These functions are typically categorized into saturated and unsaturated activation functions. In the early stages, neural networks predominantly utilized saturated activation functions, such as the sigmoid or tanh functions. However, due to the gradient vanishing problem associated with these functions, the models often struggled to converge, limiting their fitting ability. To address this issue, numerous unsaturated activation functions have emerged in recent years. One of the most popular unsaturated activation functions is the *ReLU* function [26], which is easy to implement and is computationally efficient. The derivative of the *ReLU* function is 1 for all positive inputs, effectively mitigating the problems of gradient vanishing and gradient explosion. The *ReLU* function is defined by Equation (1).

(1) $$ReLU(x)=\left\{\begin{array}{cc} 0 & x\leq 0\\ x & x>0 \end{array}\right.$$

Although the *ReLU* function provides excellent performance for neural networks, its constant derivative of 0 for negative inputs can lead to some neurons not being updated, resulting in neuron death. This phenomenon, in turn, affects the overall performance of the model. To address this issue, the *ELU* activation function [27] was introduced. The *ELU* function is defined by Equation (2).

(2) $$ELU(x)=\left\{\begin{array}{l} x\;x>0\\ \alpha (e^{x}-1)\;x\leq 0 \end{array}\right.$$

Compared to the *ReLU* function, the *ELU* function introduces a varying gradient for negative inputs. It retains the advantages of the *ReLU* function while addressing the issue of neuron death. In this study, the parameter α in the *ELU* function is set to 1. Unlike the aforementioned segmented functions, which are not smooth at input 0, the *Softplus* function [28] remains smooth at the origin and is defined by Equation (3).

(3) $$Softplus(x)=\ln(1+e^{x})$$

The *SiLU* function [29] retains the advantages of the *ELU* function and outperforms *ReLU* in deep models. Its functional expression is provided in Equation (4).

(4) $$SiLU(x)=\frac{x}{1+e^{-x}}$$

The *SiLU* function has garnered widespread attention for its excellent performance, inspiring some scholars to propose the *Mish* function [30]. Its calculation formula is given by Equation (5).

(5) $$Mish(x)=x\cdot \tanh(\ln(1+e^{x}))$$

**Figure 4:**
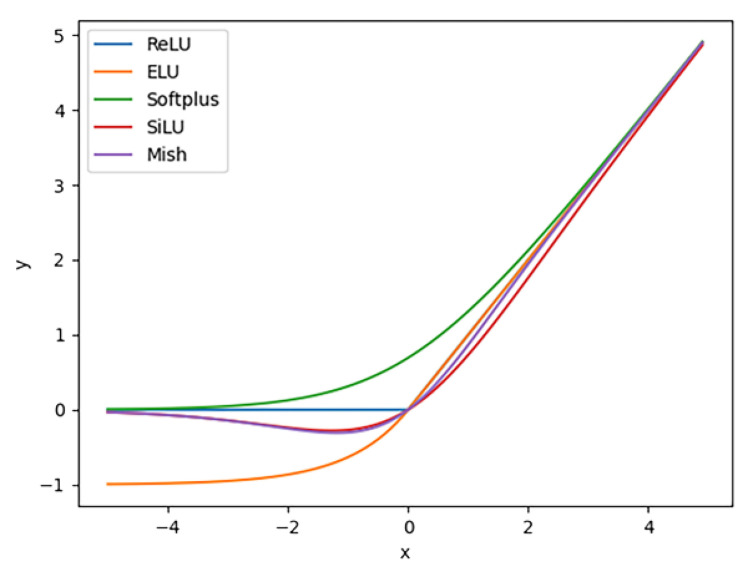
Curves of five activation functions.

Figure 4 illustrates the function curves for different activation functions. It can be observed from the figure that, in the region where the input is close to 0, the *Mish* function exhibits a larger absolute output value compared to the *SiLU* function, although their overall output values are relatively similar.

**Figure 5:**
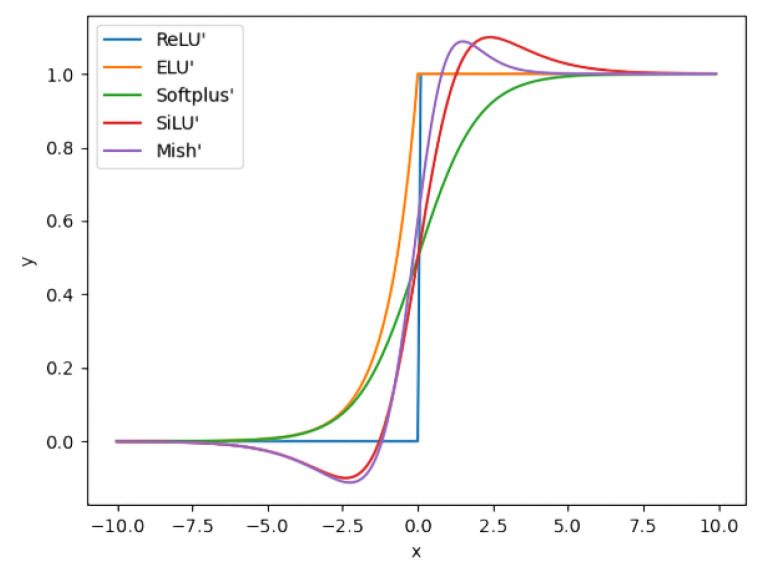
Curves of the derivatives of five activation functions.

The derivative curves of different activation functions are illustrated in Figure 5. The derivatives of the *SiLU* and *Mish* functions exhibit smoother transitions around input 0 compared to those of the *ReLU* family functions. The gradient of the *Softplus* function changes more gradually.

## 3. Results and Discussion

### 3.1. Model Validation and Testing

During the training process, the model’s performance was validated using images from the validation set at the end of each epoch. To prevent overfitting and ensure generalization, the model’s performance was further evaluated using the test set. In the prediction phase, three types of outcomes are generated for each casting defect target: true positive (*TP*), false positive (*FP*), and false negative (*FN*). Specifically, *TP* denotes correctly predicted positive samples with casting defects, *FP* denotes incorrectly predicted positive samples, and *FN* denotes incorrectly predicted negative samples. The model’s target detection performance is typically assessed using the mean average precision (*mAP*) value, which measures the overall detection performance across all target categories. The *mAP* is calculated by Equation (6).

(6) $$mAP=\frac{\sum_{i=1}^{K}AP_{i}}{K}$$

where *K* is the number of categories of the detected targets and denotes the area under the precision–recall curve. *Precision* and *recall* are calculated by Equation (7) and Equation (8), respectively.

(7) $$Precision=\frac{TP}{TP+FP}$$

(8) $$Recall=\frac{TP}{TP+FN}$$

**Figure 6:**
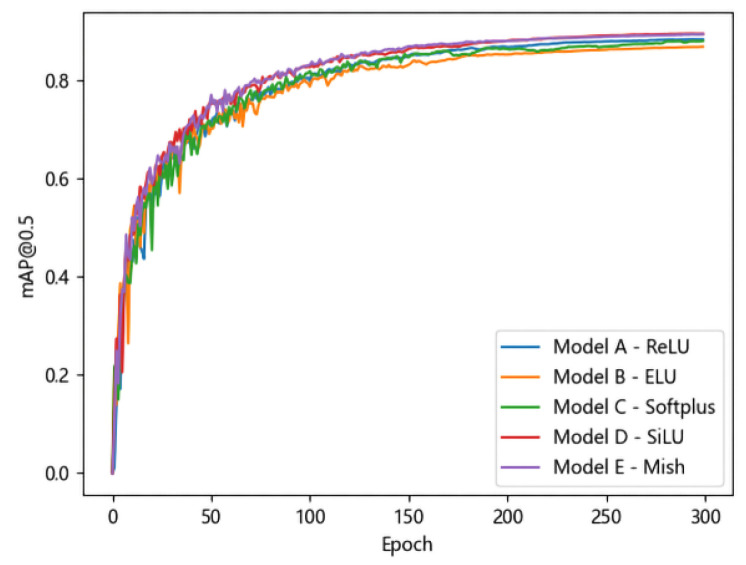
Model’s mAP@0.5 on the validation set.

The model was trained for a total of 300 epochs, with the batch size set to 16. The variation in the mAP@0.5 values of the five models on the validation set with respect to the number of training epochs is shown in Figure 6. From Figure 6, it can be observed that the *SiLU* and *Mish* functions exhibit similar performances and consistently outperform the other activation functions throughout the training process. Additionally, their curves reach a steady state earlier, meaning that the mAP@0.5 values stabilize more rapidly as the number of training epochs increases. The *ReLU* and *Softplus* functions perform similarly but achieve lower mAP@0.5 values compared to *SiLU* and *Mish*. Despite introducing a gradient for negative inputs, the *ELU* function remains less accurate than the *ReLU* function in detection tasks. The experimental results demonstrate that the *SiLU* and *Mish* functions possess superior fitting capabilities for casting defect detection and are thus more suitable for this application. The computational complexity of the *Mish* activation function is higher than that of the *SiLU* activation function, yet its performance in detecting casting defects is superior. When the device has sufficient computing power, employing the *Mish* activation function can yield better results.

To further validate the accuracy of the five models, this study evaluated each model on the test set. In addition to mAP@0.5, the study also utilized AP and mAP@0.5:0.95 values for each defect class to assess model performance.

Table 1 presents the performance of the five activation functions on the test set. The results are generally consistent with those from the validation set, except for some differences observed in the *ELU* and *Softplus* activation functions. The *Softplus* activation function yields lower values for each data point in the test set, suggesting that it exhibits relatively poor generalization performance. The experimental results for the *SiLU* and *Mish* activation functions remain closely aligned, with the *Mish* function showing slightly higher performance overall, except for the AP value of pore detection. This suggests that the *Mish* function’s larger absolute output near zero enhances the model’s mapping of casting target features, thereby improving target discrimination. The experimental results demonstrate that the *Mish* activation function exhibits superior performance in casting defect detection.

To evaluate the stability of the object detection model under different sample configurations, we employed the Bootstrap resampling method to construct multiple test sets. Random sampling with replacement was conducted from the original test set, and each sampling generated a test subset of the same size as the original test set. This procedure was repeated 1000 times to construct 1000 distinct test subsets. Subsequently, each test subset was evaluated, and the AP for each category as well as their mAP values were statistically analyzed. Additionally, the 95% confidence intervals (CI) were calculated, as presented in Table 2.

As shown in Table 2, the *SiLU* and *Mish* activation functions demonstrated superior overall detection performance, with 95% confidence intervals of (0.880, 0.920) and (0.877, 0.919), respectively. These intervals exhibit higher upper limits compared to those of other activation functions, suggesting that their performance remains robust across varying training samples. The *ReLU* activation function shows a narrower fluctuation range; however, both its upper and lower bounds of the 95% confidence interval are relatively lower. In contrast, the *Softplus* activation function exhibits the widest 95% confidence interval for overall detection performance. Notably, when detecting loose defects, its 95% confidence interval ranges from 0.787 to 0.962, indicating considerable variability in its performance for this specific task.

As shown in Table 1, different activation functions led to varying model accuracies on both the validation and test sets. Given that the architecture of the five models is identical, the differences in accuracy are primarily attributed to the weights of each feature layer. However, neural network weights are typically high-dimensional arrays that lack intuitive interpretability when viewed directly. The forward computation in the feature layers transforms the input casting image information by recalculating the feature maps through the weights, gradually extracting casting defect features to achieve accurate defect recognition. Therefore, this study conducted statistical analysis on the mean and variance of different feature layers to compare and analyze the impact of the five models on accuracy.

### 3.2. Mean and Variance of Feature Layers

To quantitatively analyze the effects of the five activation functions on each feature layer, this study sets up three groups of experiments to compute the mean and variance of the feature layers corresponding to the trunk part and the detection head. The mean value represents the average value of the casting defect image across different feature layers, whereas the variance indicates the extent of data fluctuation. The horizontal coordinate value -1 represents the normalized mean and variance of the input original image, to highlight the influence of the activation function on different defects and avoid additional impacts caused by excessive background areas. For each experiment group, the input image contains only one type of defect—porosity, inclusions, or looseness—and includes only a single instance of that defect, with an image size of 100 × 100 pixels.

**Figure 7:**
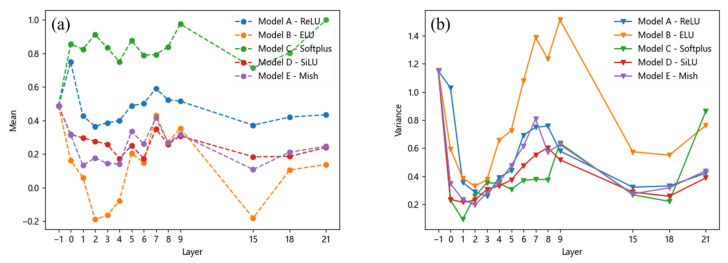
Mean and variance of the pore: (**a**) mean, (**b**) variance.

For porosity, the mean and variance vary with the feature layer, as shown in Figure 7. Under the *SiLU* and *Mish* activation functions, the mean values are close and fall within an appropriate range. Although the *ReLU* activation function exhibits a similar trend, its mean values are higher than those of the *SiLU* and *Mish* functions. This can be attributed to the absence of negative values in the output of the *ReLU* function, as illustrated by the activation function curves in Figure 4. Additionally, the *Softplus* activation function also yields higher mean values for pores compared to the *ReLU* function, further supporting this observation. However, the mean values of pores under the *ELU* activation function exhibit a significant change.

**Figure 8:**
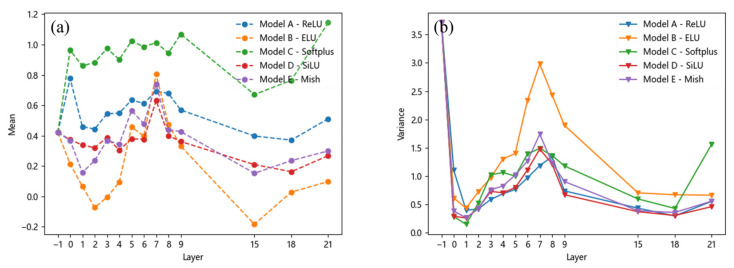
Mean and variance of the inclusion: (**a**) mean, (**b**) variance.

**Figure 9:**
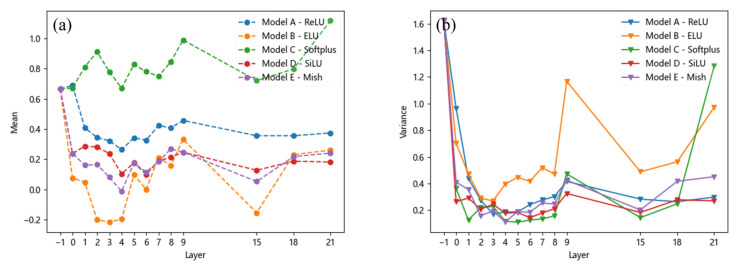
Mean and variance of the looseness: (**a**) mean, (**b**) variance.

In contrast, there is no significant difference in the variance of stomata across all the models except for the *ELU* activation function. This suggests that the *ELU* function’s larger derivative in the left half leads to more frequent weight updates during training, resulting in greater variations in feature map eigenvalues and thus higher variance. Similar conclusions can be drawn for inclusion and looseness, as shown in Figure 8 and Figure 9, where their mean and variance also change with the feature layer. Overall, it can be observed that although the variance of different defects under the *ReLU* and *Softplus* activation functions yields results similar to those of the *SiLU* and *Mish* activation functions, there are significant differences in their mean values. The superior performance of the *SiLU* and *Mish* activation functions is primarily attributed to their ability to introduce negative values in the output, thereby increasing the disparity of certain output values. Consequently, their mean values are relatively lower. The *ELU* activation function exhibits a similar effect; however, its large variance suggests that the model’s output lacks stability, which may hinder effective model training.

### 3.3. Key Regions in Radiographic Images

From the test results in Table 1, it is evident that the *Mish* activation function achieves higher detection accuracy for casting defects compared to other activation functions. This indicates that Model E is more sensitive to casting defects and can better focus on different types of defects.

**Figure 10:**
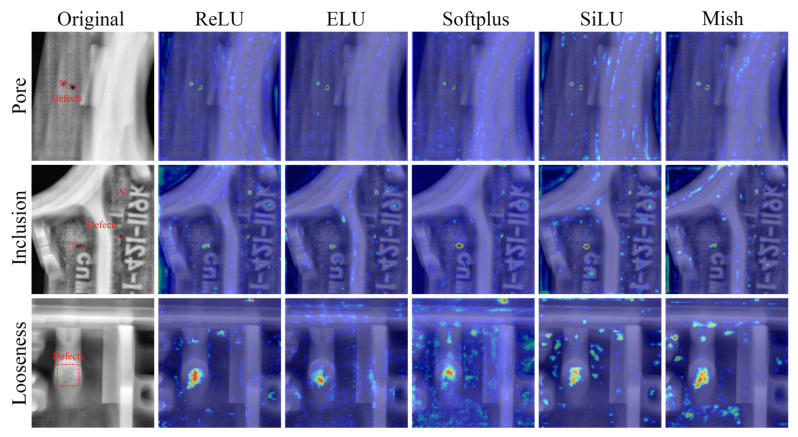
Grad-CAMs of three types of defects under five models.

To further analyze the performance differences among the five models, this study calculated gradient class activation mappings (Grad-CAM). Given that defective targets in castings are generally small, larger-sized feature maps in the network structure have higher attention to small targets. Therefore, Grad-CAM was computed for 15 layers, as shown in Figure 10. In Figure 10, the red regions indicate areas where the model pays more attention, while the blue regions indicate areas receiving less attention. From Figure 10, it can be observed that all five models effectively focus on the three types of defective regions in castings. However, compared to the other models, the *SiLU* and *Mish* activation functions exhibit more secondary regions of interest for porosity and inclusion defects, which typically occur at grayscale changes in the original casting images. Casting defects are precisely reflected as grayscale changes in the images. This suggests that Models D and E are more sensitive to casting defects with subtle grayscale changes, leading to higher detection accuracy compared to the other models.

In particular, for loose defects, the *Softplus* activation function exhibits too many secondary regions of interest, making it difficult for the model to distinguish between the background and the target. This explains why Model C has the lowest detection accuracy for loose defects, as shown in Table 1. By analyzing the images of the *Softplus* activation function, it can be observed that the differences between the output values after input mapping by the *Softplus* activation function are relatively small. Consequently, the recognition ability for loose defects with minor changes in background gray levels is limited, leading to excessive focus on the background.

The above results demonstrate that using the *Mish* activation function enables the model to effectively focus on regions with grayscale changes in the casting image while paying less attention to background areas. This enhancement improves the model’s ability to detect casting defects.

**Figure 11:**
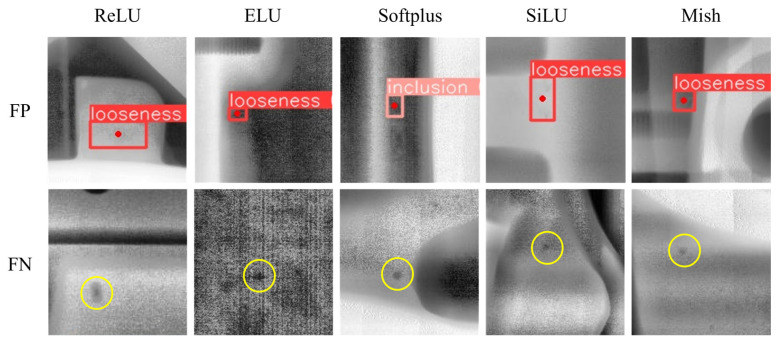
Examples of FP and FN of five activation functions.

To gain a more intuitive understanding of the impact of defect confusion with the background, Figure 11 presents examples of false positives (FPs) and false negatives (FNs) under five activation functions. In the figure, FP refers to cases where the model incorrectly classifies the background as a defect target, while FN refers to cases where the model misclassifies a defect target as the background. Both types of errors are highlighted with yellow circles. For FPs, due to the relatively small gray-level variation in loose defects, the model is more likely to confuse loose defects with the background. For FNs, the model tends to miss detections, particularly for small-sized pores or inclusions.

## 4. Conclusions

This paper investigates the impact of activation functions in convolutional neural networks (CNNs) on casting defect detection. By analyzing the mean and variance of feature maps across different layers and the gradient class activation mapping (Grad-CAM) of feature maps, the following three conclusions are drawn:

1. The *Mish* activation function achieves optimal casting defect detection performance compared to other experimental activation functions, with an mAP@0.5 value of 90.1% on the used dataset, while the *Softplus* activation function only achieves an mAP@0.5 value of 86.7%.

2. For cast DR images, the *Mish* activation function maintains the mean and variance of feature maps within a suitable range, preventing values from becoming overly concentrated or dispersed, thereby avoiding negative impacts on model performance.

3. The gradient class activation mapping results indicate that the *Mish* activation function enhances the model’s focus on regions with grayscale variations in cast DR images, thereby improving detection accuracy.

Although the *Mish* activation function exhibits the highest casting defect detection performance in this study, Figure 10 shows that the model also pays some attention to background regions with grayscale variations. This suggests that the model struggles to effectively discriminate between background and casting defects, leading to potential false detections when the contrast of casting defects is low. Future work could explore specific modules to reduce the model’s focus on non-defective regions with grayscale variations.

## Figures and Tables

**Table 1:** AP and mAP of five models.

Activation Function	AP/%			mAP@0.5/%	mAP@0.5:0.95/%
	Pore	Inclusion	Looseness		
Rectified Linear Unit (*ReLU*)	89.5	84.5	91.9	88.6	58.0
Exponential Linear Units (*ELU*)	89.1	83.6	89.0	87.2	56.4
*Softplus*	89.0	84.0	87.1	86.7	56.2
Sigmoid Linear Unit (*SiLU*)	90.9	85.9	92.8	89.9	60.4
*Mish*	90.9	86.0	93.3	90.1	60.6

**Table 2:** Ninety-five percent CI of five activation functions.

Activation Function	95% CI			
	Pore AP	Inclusion AP	Looseness AP	mAP@0.5
*ReLU*	(0.861, 0.924)	(0.806, 0.878)	(0.892, 0.966)	(0.869, 0.907)
*ELU*	(0.861, 0.922)	(0.802, 0.868)	(0.846, 0.962)	(0.855, 0.906)
*Softplus*	(0.864, 0.918)	(0.798, 0.871)	(0.787, 0.962)	(0.845, 0.905)
*SiLU*	(0.887, 0.941)	(0.819, 0.891)	(0.894, 0.964)	(0.880, 0.920)
*Mish*	(0.874, 0.935)	(0.815, 0.890)	(0.906, 0.961)	(0.877, 0.919)

## Data Availability

The original contributions presented in this study are included in the article. Further inquiries can be directed to the corresponding author.
